# The association between air pollution exposure and childhood cancer: a scoping review about the challenges in epidemiological studies

**DOI:** 10.1186/s12889-025-25754-x

**Published:** 2025-12-03

**Authors:** Hiba Oqba, Lorena Cascant Ortolano, Susanne Singer, Maria Blettner, Emilio Gianicolo

**Affiliations:** 1 Institute for Medical Biostatistics, Epidemiology and Informatics (IMBEI), Division Epidemiology and Health Services Research, University Medical Center of the Johannes Gutenberg University, Mainz, Germany; 2 Departmental Library for the University Medical Center, Johannes Gutenberg University, Mainz, Germany; 3 Quality of Life in Oncology, Comprehensive Cancer Center Mecklenburg-Vorpommern, University Hospital Rostock, Rostock, Germany

**Keywords:** Methodological challenges, Childhood cancer, Air pollution, Epidemiological study

## Abstract

**Background and objective:**

Although several studies have investigated the association between various air pollutants and the risk of childhood cancer, particularly leukemia, results are still inconclusive. This scoping review aims to identify and discuss the main methodological challenges observed in publications addressing this topic.

**Methodology:**

A literature search was conducted in two databases (MEDLINE and Web of Science), focusing on epidemiological studies that examine the association between air pollution exposure and childhood cancer with publication dates ranging from 2009 to 2024. To ensure completeness, we conducted citation tracking and a supplementary Google Scholar search. Relevant publications were evaluated regarding different methodological aspects.

**Results:**

Two independent reviewers screened 1683 abstracts and finally evaluated 32 full-text articles based on predefined criteria. Additionally, two cohort studies were included from citation tracking and one case-control study from Google Scholar. Twenty-three studies investigated the association between air pollutants and childhood cancer, 19 of them were case-control studies. This design poses a risk of recall and selection biases. Many studies face challenges such as inconsistent air pollution measurements, varying exposure time frames, and different data sources. Furthermore, many studies under-adjust important confounders like socioeconomic status, radiation levels, urbanization, and parental smoking habits. These issues limit internal validity and make it difficult to draw firm conclusions about the association.

**Conclusion:**

Further studies should use more robust methodologies including improved exposure assessment, larger study populations, and advanced statistics, to strengthen the association between air pollution and childhood cancer and inform public health policies.

## Background

Air pollution is a huge global health challenge. Evidence shows that adverse health effects exist even at low air pollutant concentrations. Ambient air pollution causes strokes, cardiovascular illnesses, lung carcinoma, and both short-term and long-term respiratory conditions [1]. Measurement of exposure is difficult, due to long sampling duration, costly and highly sophisticated equipment with technical expertise needed for ground-level monitoring [2].

Additionally, based on sufficient evidence for lung cancer and positive association with bladder cancer, mainly from studies in adults, the International Agency for Research on Cancer (IARC) in 2013, has classified outdoor air pollution as well as particulate matter (PM), as Group 1 (carcinogenic for humans) [3, 4]. Similarly, benzene has been deemed by IARC as carcinogenic for humans, and even at very low exposure levels has been linked to the haematological and genotoxic effects particularly among susceptible groups like children [5, 6].

Children are more susceptible than adults to air pollutants. One of the reasons is that children spend more time outside than adults, so they are more exposed to air pollutants. Additionally, children inhale more air because they breathe more rapidly, and therefore, they have a higher respiratory rate. Furthermore, children’s developmental stage at the time of exposure plays a significant role, either during or after the organogenesis is complete. In the first stage, it is more likely this leads to permanent structural changes while in the second stage it can lead to functional changes. At birth, the immune, respiratory, and central nervous systems are still immature and need a prolonged period of postnatal maturation. Consequently, these organ systems are endangered to postnatal exposures [7].

In addition to postnatal exposures, prenatal exposure is considered a critical period in pediatric carcinogenesis. In fetal developmental periods, there are rapid cellular proliferation, immature detoxification systems along with incomplete DNA repair capacity, this increases the vulnerability of the fetus to various environmental pollutants [8]. During pregnancy, various air pollution particles can penetrate the placenta, lead to genetic and epigenetic alterations, subsequently increasing the susceptibility to develop adverse health effects in neonatal and childhood periods [9]. Moreover, several epidemiological studies have suggested that prenatal exposure to traffic-related air pollution is associated with higher risk of early childhood cancers [10]. Schüz, J. and Erdmann, F. (2016) did a review of environmental risk factors highlighting that parental and in-utero exposures contributed to childhood leukemia risk [11]. In 2018, Greaves, M suggested two-hit model, specifically for childhood B-cell precursor acute lymphoblastic leukemia (BCP-ALL), in which prenatal genetic changes act as first hit and then postnatal exposures, such as delayed infections in his study consider as the second hit that together triggers leukemogenesis [12]. This model is specific to BCP-ALL and does not apply to all childhood cancers. However, the conceptual distinction between prenatal and postnatal exposure highlights the broader importance of considering multiple developmental windows when investigating childhood cancer etiology and when designing epidemiological studies.

Childhood cancers are diverse and show a remarkable variety of tumor types, including several groups that are mostly unique to children. The most common types of childhood cancers include leukemia, brain cancers, lymphomas and solid tumors, such as neuroblastoma and Wilms tumors [13, 14]. Leukemia is the most common cancer being diagnosed in children, primarily in the form of acute lymphoblastic leukemia (ALL), then acute myeloid leukemia (AML), whereas chronic leukemia is rare in children [15].

According to the World Health Organization (WHO), the global estimate of cancer incidence in children aged between 0 and 19 years is approximately 400,000 per year [16]. Researchers have investigated a wide range of potential risk factors for childhood cancer, including pregnancy-related variables, infections in early life, environmental exposures, parental lifestyles and occupational exposures of the parents. However, only a few known risk factors explain a small proportion of cases (< 10% of all cases), including exposure to high-dose ionizing radiation, certain chromosomal and genetic conditions, low or high birth weight, and advanced maternal age at the child’s birth. The results of epidemiological studies focusing on the relation between air pollutants and childhood cancer are still inconclusive [17, 18]. One reason is the presence of methodological challenges, such as recall and selection biases, difficulties in exposure assessment across critical periods, limitations in data quality for outcome measurement, challenges in control selection. Potential confounders such as socioeconomic status (SES), urbanization level, and parental smoking status, may distort observed associations. Additional environmental exposure, such as background gamma radiation (a form of ionizing radiation), may also act as independent risk factors or interacting exposures, rather than effect modifiers that bias associations. To date, no scoping review has systematically addressed and summarized the methodological challenges in studying the association between air pollution exposure and childhood cancer. Previous reviews have mainly focused on summarizing the findings rather than critically discussing the limitations that impede the causal inference. Our review aims to fill this gap by mapping the main methodological challenges faced in this research field. By doing so, we provide a foundation to improve the design of future epidemiological studies and to contribute to more reliable evidence for policymakers and public health interventions.

## Methodology

### Search strategy

The search strategy was developed in consultation with a librarian (LCS) at the University Medical Centre of the Johannes Gutenberg University Mainz. This review was conducted in accordance with the PRISMA Extension for Scoping Reviews (PRISMA-ScR) guideline [19].

A comprehensive literature search was conducted to include eligible papers published between January 1 st, 2009 until June 5th, 2024 in MEDLINE (via PubMed) and Web of Science Core Collection (Clarivate). We chose 2009 as the starting point for our scoping review because it marks the beginning of prominent methodological advancements in air pollution exposure measurement and the increased availability of high-quality environmental data in recent years. Since then, new tools as satellite-based remote sensing and land-use regression models have provided exposure estimates with greater spatial and temporal resolution than previously available [20, 21]. These methodological improvements justify focusing on studies from 2009 onward, as this year represents a turning point for data availability and epidemiological methodological advancement [22, 23].

A combination of Medical Subject Heading (MeSH) and free text terms for “neoplasms” and “air pollution” were identified using the following automation tools: ‘The Yale MeSH Analyzer’ [24] and ‘PubMed PubReminer’ [25]. Validated filters for identifying case-control studies and cohort studies were applied in MEDLINE [26]. A validated filter for retrieving pediatric studies in MEDLINE was applied and adapted for Web of Science [27]. No restrictions on language were used, all studies obtained, regardless of publication language, were included in screening. Because the search strategy applied filters targeting only primary epidemiological study designs (case-control and cohort), systematic reviews and meta-analyses were not retrieved through the database searches. To enhance completeness, citation tracking was conducted; the reference lists of all studies included after full-text screening were manually reviewed to identify additional eligible primary studies. When systematic reviews and meta-analysis were encountered during this process, their reference lists were also screened to ensure that no relevant original studies were missed. A supplementary search in Google Scholar was performed to capture additional grey literature and potentially missed primary studies. Duplicates were removed using the tool “Deduplicator” [28]. The search algorithms for all databases and complete search syntax are available in the + Appendix to ensure reproducibility.

### Eligibility criteria (PICOS Framework)

The following eligibility criteria were applied, guided by the PICOS framework:

- Population (P): Children aged 0–18 years. In accordance with the United Nations Convention on the Rights of the Child (UNCRC), childhood was defined as “every human being below the age of 18 years”. Article 1 of the Convention on the Rights of the Child (CRC) considers 18 years as the standard endpoint of childhood, providing an internationally recognized cut-off for our eligibility criteria [29]. Studies with broader age ranges were included only if they reported data separately for the 0–18-year-old population.
- Exposure (I): Exposure to outdoor air pollutants (e.g., PM_2.5_, PM_10_, NO_2_, O_3_, Benzene, traffic-related pollution).
- Comparator (C): Children without documented exposure, as defined within each study.
- Outcome (O): Childhood cancers (e.g., leukemia, brain tumors, lymphomas, neuroblastoma, Wilms tumor).
- Study Design (S): Observational epidemiological studies (case-control and cohort studies).

#### Inclusion criteria

1. Studies investigating the association between exposure to air pollutants and childhood cancer.
2. Articles published between January 1 st, 2009 and June 5th, 2024.
3. Studies including primary research (case-control or cohort studies).

#### Exclusion criteria

1. Studies including participants older than 18 years without separate reporting for children.
2. Studies focusing exclusively on chemical pollutants other than air pollutants.
3. Ecological studies (without individual-level data were excluded), bibliometric analyses, reviews, commentaries, conference abstracts, and case reports.
4. Systematic reviews and meta-analyses as (Filippini et al., 2019, and Gong et al., 2019), since the aim of this scoping review was to identify methodological challenges in original primary epidemiological studies (case-control and cohort studies), rather than to summarize existing evidence [30, 31].

### Manuscript screening and data abstraction

All titles and abstracts were screened by one reviewer (HO) using “Screenatron” [32]. Full texts of all eligible studies were acquired and then evaluated by two independent reviewers (HO, RH). Any disagreement was resolved by discussion between the two reviewers resulting in a consensus decision.

### Data extraction

For each included article, we extracted the following information: authors, year of publication, country, study design, study period, age range, number of participants, matching and adjustment factors, cancer type, air pollutant type, assessment of the exposure, challenges in methodology, results of the study, strengths, and limitations. To address the challenges, we used the framework outlined in the 4th edition of “Modern Epidemiology” by Rothman et al. (2021) [33] as the guiding scheme for data extraction and methodological evaluation.

## Results

**Fig. 1:**
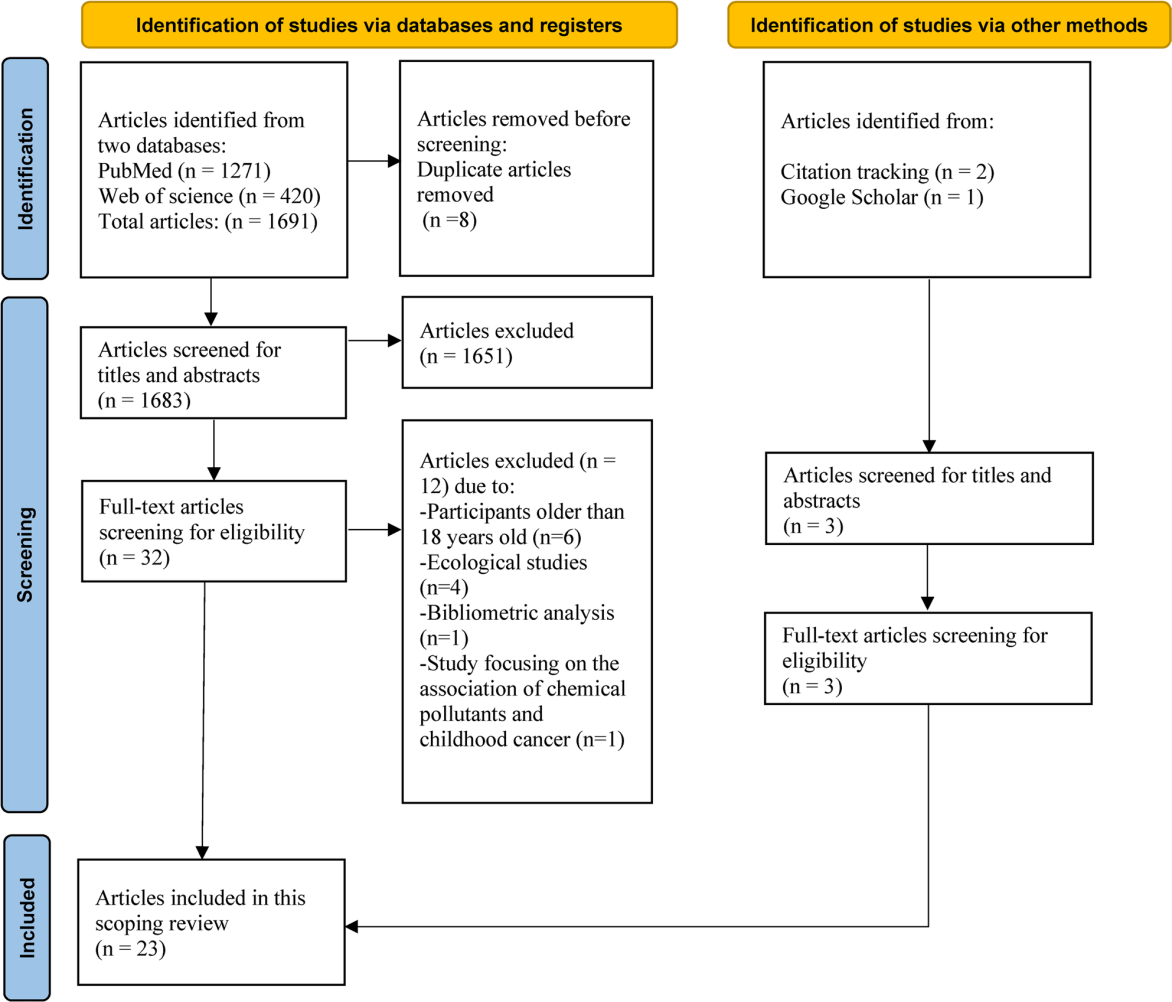
Flowchart showing the process for scoping review

The databases queries in MEDLINE via PubMed (*n* = 1271), Web of Science (*n* = 420) and the citation searching (*n* = 2), Google Scholar (*n* = 1), yielded 1694 results in total, of which 1686 remained after checking for duplicates. After title and abstract screening, 32 studies remained for full-text review. Of these, six were removed because they included participants older than 18 years old [34–39]. Four were removed because they were ecological studies [40–43]. One was removed because it was a bibliometric analysis [44]. One was removed because it studied the residential proximity to industrial and urban areas mainly focusing on the association of chemical substance and childhood cancer [45], leaving 23 studies eligible for data extraction in this review. The literature selection process is presented in Fig. 1 below.

### Study characteristics

Of the included articles, 19 were case-control studies and four were cohort studies. The study periods ranged from 1 to 30 years. Geographically, seven studies were conducted in the USA, four in Italy, three in France, three in Switzerland, one in Denmark, one in Canada, one in South-Korea, two in Iran and one in Taiwan. Table 1 below summarizes the main characteristics of the 23 studies.

**Table 1:** Characteristics of studies included in the scoping review

References	Country	Study period	Age	Participant numbers	Cancer type	Pollutant type	Matching factors	Adjustment factors	Assessment of exposure	Result of study
Williams et al., 2024	Texas, USA	1995–2011	0–16 years	6101/109,762	childhood cancer	PM_2.5_	sex and birth year	birth year, sex, maternal race/Ethnicity (White, Hispanic, Black, Asian), socioeconomic status	They use satellite data from NASA (MODIS, MISR, and SeaWiFS) to create the PM_2.5_ model, and used annual mean PM_2.5_ values for analysis and assigning them based on the child’s birth year.	Increase the risk of childhood cancers (lymphoid leukemias, Hodgkin lymphomas, non-Hodgkin lymphomas, and ependymoma) is linked to PM2.5 exposures in early life. Decrease the risk of ependymoma and medulloblastoma and increase risk of malignant melanoma and Langerhans cell histiocytosis linked to higher NDVI. Also, a statistical interaction between NDVI and PM_2.5_ for all cancers.
Norzaee et al., 2024	Tehran, Iran	2016–2021	1–15 years	856/428	leukemia and lymphoma.	proximity to land use of roads, petrol stations, power lines, and bus stations.	age, sex, family history, parental smoking.	parental smoking and child’s sex, birth year, family history of cancer.	A GIS- using circular buffers of various radii around locations like main roads, petrol stations, bus stations, and power lines. Main roads were divided into Class 1 (highways) and Class 2 (city roads). For each land use type, they used different distance thresholds.	A higher association was found between leukaemia and lymphoma and children living within 100 m of highways or petrol stations, particularly near power lines for leukaemia only.
Zhong et al., 2023	California, USA	2000–2015	0–14 years	2782/139,100	ALL	PM_2.5_	year of birth	Adjusted for neighborhood poverty level, sex, race/ethnicity, birthweight, mode of delivery, maternal age, maternal education, mother’s place of birth, complications during pregnancy and paternal age.	An ensemble-based model was used to assign average PM_2.5_ levels from birth until diagnosis. This model generated daily PM_2.5_ concentrations at a 1-km resolution, with validation of an R² of 0.802 for the Pacific region, estimated by integrating data from a chemical transport model, land use, population density, weather patterns, and satellite derived aerosol measurements.	Higher levels of ALAN were linked to an increased risk of ALL in Hispanic children. Whereas a borderline association between PM_2.5_ levels and ALL risk in non-Hispanic children.
Malavolti et al., 2023	Northern Italy	1998–2019	0–14 years	182/726	Leukeamias	Residential proximity to petrol stations	sex, year of birth, province of residence during the year of diagnosis.	PM_10_ concentration, magnetic field ELF-MF from power lines, socio-demographic variables maternal age/ethnicity, parental annual income for index year, urban and indoor transformer station	Petrol station exposure was assessed by measuring the distance from homes to the nearest station, divided into categories with the following cut points: 50, 200, 500, 1000 and over 1000 m. Also, estimating the annual fuel sales of stations within 1000 m of residences.	Higher risk of leukemia, especially the ALL subtype is stronger in children (age ≥ 5 years) living within 50 m of a petrol Proximity to petrol stations with high refueling activity also increased leukemia risk, though results were imprecise.
Kreis et al., 2022	Switzerland	1990–2015	< 16 years	2,960/296,000	childhood cancer	NO_2_ and benzene	Linkage with the SNC was done (sex, date of birth) and residential details (geocode, municipality, nationalty).	Neighbourhood socio-economic position, level of urbanization, and background ionizing radiation.	NO_2_ exposure at children’s residences was modeled using dispersion modelling and LUR. For benzene, only a 200 m resolution dispersion model (PolluMap) was used due to limited data, with exposures taken directly from its 1990/2010 estimates.	NO_2_ increased risk of AML and some CNS tumors but not ALL. Benzene exposure also increased risks for lymphomas, soft-tissue sarcomas, and malignant bone tumor, and weaker links to leukemia.
Lee et al., 2022	Korea	2002–2012	5–15 years	1,261,855	Childhood cancer	PM_2.5_ and PM_10_	NA	Adjusted for sex, birth year, SES (insurance type, city of residence at birth, family) area-level smoking rate.	Using AirKorea’s monthly mean air pollutants (PM_10_, PM_2.5_, NO_2_, SO_2_, CO, and O_3_) at the city, county, and district levels in Korea. PM_2.5_ before 2015 (when monitoring began), they estimated levels using an air-quality model.	In a large cohort, PM_2.5_ exposure was linked to childhood cancer risk, mainly for lymphoid leukemia.
Mazzei et al., 2022	Switzerland	1985–2015	0–15 years	6129/61,290	leukeamia and CNS tumors	Benzene	age, sex, and census corresponding to year of diagnosis of the index cases.	Ambient NO_2_, highways proximity, urbanization level, and cantonal cancer registry availability.	Proximity to petrol stations was used as a proxy for exposure, calculated from residences to the nearest station using Swiss Business Censuses geocodes.	living near petrol stations was weakly associated with increased risk of all childhood cancer, leukemia, and CNS tumors.
Lavigne et al., 2020	Toronto, Canada	1998–2017	0–14 years	653,702 singleton live births	childhood cancer	Ambient ultrafine particles (< 0.1 μm) (UFPs)	NA	Adjusted for distributed lag exposures of PM_2.5_ and NO_2_, during the pregnancy and after Birth. Maternal demographics (age at delivery, parity, year of birth, smoking), infant sex, Area-level socioeconomic confounders (income, ethnicity, education)	The LUR model for UFPs in Toronto, explained 67% of UFP variation and provided spatial and temporal exposure estimates, was developed using mobile monitoring. Similar methods were applied for NO_2_ and PM_2.5_ exposures during pregnancy and childhood.	They linked exposure to UFPs during the first trimester with an increased overall cancer incidence in children diagnosed before age six, a linear relationship found after adjustment for confounders. No association found for specific cancer subtypes.
Peckham-Gregory et al., 2019	Texas, USA	1995–2011	0–16 years	2030/20,300	acute leukemias	maternal residential proximity to major roadways	birth year	birth year, maternal race/ethnicity, maternal education, area-level poverty, birth weight in grams and child sex.	Maternal proximity to major roadways was assessed using three measures: distance to the nearest major roadway, presence of a major roadway within 500 m, and roadway density within 500 m, using the Texas roadway network StratMap. Also, GIS were used.	The study found no strong association between maternal proximity to major roadways and the risk of childhood acute leukemia (ALL or AML), with risk estimates close to null, indicating that living near major roadways does not significantly increase the risk of acute leukemia in children.
Seifi et al., 2019	Tehran, Iran	2007–2009	1–15 years	161/761	Leukemia, sarcoma, neuroblastoma, retinoblastoma, germ cell tumors	Annual mean PM₁₀, NO₂, SO₂ assigned to geocoded residential addresses using LUR model	Not found	Not found	Air pollutants were estimated with a 2010 GIS-based LUR for all three pollutants at each residence.	Children with cancer had a higher exposure to PM_10_ and NO_2_ than controls, and only PM_10_ showed a significant positive association with childhood cancer risk, while NO_2_ was positive but not significant and SO_2_ showed no association.
Raaschou-Nielsen et al., 2018	Denmark	1968–1991	0–14 years	1989/5,506	leukemia, lymphoma and CNS tumors	Benzene	sex, age and calendar time	degree of urban development, geographical region, type of residence, EMFs, mother’s age and birth order	Benzene exposure at residences was calculated using the Operational Street Pollution Model, with exposure summarized as “exposure level x time” during pregnancy and childhood. The time-window for exposure was the same for cases and controls.	Higher childhood benzene exposure was linked to an increased risk of AML, but not ALL. There was also a varied risk for CNS tumors, with lower risk for ependymoma and higher risk for medulloblastoma.
von Ehrenstein et al., 2016	California, USA	1990–2007	0–5 years	183/30,569	Brain tumors: medulloblastoma, central nervous system primitive neuro ectodermal tumor (PNET), and astrocytoma	ambient air toxics (26 probable carcinogenic toxics) including: 1,3-Butadiene, Benzene	Birth year	Maternal, child, and SE factors (including birth year, maternal age, race/ethnicity, place of birth, education, insurance type) and did not change the estimates of interest > 3%.	Since 1990, the California Air Resources Board (CARB) has collected 24-hour air samples every 12 days from 31 monitors across the state near highways, industrial, and agricultural areas, focusing on 42 substances categorized as carcinogens by the IARC.	Prenatal and early life exposures to industrial and road traffic air toxicity may increase the risk of PNET and medulloblastoma in young children ≤ 6 years), with limited evidence for increased astrocytoma.
Magnani et al., 2016	Italy	1998–2001	0–10 years	645/980	Leukeamias(ALL and AnLL)	Road Traffic Pollution	birth date +/−15d), gender and region.	age, sex, region, parental education, parental smoking.	Traffic exposure data was collected via questionnaires and geocoded addresses, within 500 m as metric. Detailed methodology on geocoding, dispersion models and LUR was described in Badaloni et al.	The SETIL study linked traffic pollutants to An increased risk of AnLL and, to a lesser extent ALL, especially near traffic lights and truck routes. The association was stronger for AnLL than for ALL.
Symanski et al., 2016	Texas, USA	1995–2011.	under 5 years	1,248/12,172	ALL	benzene, 1,3-butadiene and POM	birth month and year	matching variables and census tract (random effect)	Benzene, 1,3-butadiene, and POM exposures were assigned by linking maternal addresses to U.S. EPA NATA data at census tract level.	Early life exposure to 1,3-butadiene was linked to an increased risk of childhood leukemia, while benzene and POM showed weaker associations in co-pollutant models.
Spycher et al., 2015	Switzerland	1990–2000	less than 16 years	2,096,402	Childhood cancer	proximity to highways and traffic volume on nearest highway	Record linkage between SNC and SCCR was based on sex, date of birth, parental dates of birth, geocoded and municipal residence date and nationality	Sex, birth year, Socio-economic factors, ionising background radiation and EMFs.	Highway exposure was measured by the distance from residences to the nearest highway. For children living within 500 m. Traffic volume data and digital maps were incorporated.	Living within 100 m of highways increased risk of leukemia, especially ALL in children under 5 years old. No significant association was found with other tumors or traffic volume.
Houot et al., 2015	France	2002–2007	< 15 years	2760/30,000	Leukeamias	Benzene and NO_2_	urban unit size, median income, and blue-collar workers, high-school graduates, and homeownership.	Stratified by age groups (≤ 4, 5–9, 10–14), calendar periods; urban unit size and a commune-level deprivation index derived from Unemployment, education, occupation and income.	The study defined high traffic exposures as living within 150 m of a major road, using two indicators: proximity and road length. NO_2_ and benzene concentrations were modeled, with background benzene estimated by combining monitoring and dispersion modelling data.	A 300-meter increase in major road length within 150 m of a child’s home was significantly associated with an increased risk of AML, especially when combined with benzene exposure. No association was found with ALL and traffic-related NO_2_ was not linked to either AML or ALL.
Heck et al., 2014	California, USA	1990–2007	0–5 years	for ALL 69/2994 for AML 46/19,209	Leukeamias	Ambient air toxics from fuel combustion, including PAHs, arsenic, benzene and chloroform	year of birth	birth year, maternal race/ethnicity, child’s sex, rural/urban area of residence, and maternal age.	Exposure data for 42 IARC carcinogens came from were collected from 39 community air monitors. Total PAHs, was created by summing specific PAH values.	The 3rd trimester exposure to benzene and other toxics related to fuel combustion increased risk of ALL and AML. In the child’s first year, exposure to butadiene, ortho-xylene, and toluene increased risk for AML, while selenium increased risk for ALL. Suggesting that prenatal and early-life exposures to benzene and other chemicals linked to childhood leukemia.
Badaloni et al., 2013	Italy	1998–2001	≤ 10 years	620/957	Acute leukemias	PM_2.5_, PM_10_, NO_2_ and O_3_	birth date, gender and region of residence	age at diagnosis, gender and region of residence, paternal and maternal educational levels	Exposure at birth residence was assessed using traffic proximity to main roads and length of main roads within 100 m) and modeled pollutant concentrations (PM_2.5_ from national dispersion model and LUR models incorporating satellite data for PM_10_, NO_2_ and O_3_.	No association was found, and all Odd ratios, independent of the method of assessment and the exposure windows were close to the null value.
Ghosh et al., 2013	California, USA	1988–2008	0–5 years	4,015/80,658	ALL, AML, non-Hodgkin lymphoma, CNS tumors.	nitric oxide, nitrogen dioxide, and nitrogen oxides	birth year and sex	Adjusted for maternal demographics (age, race/ethnicity, educational level, parity) insurance type, Census-based socioeconomic status, child’s sex and birth year.	A LUR model for Los Angeles County estimated vehicle exhaust exposure with high accuracy explaining 81%, 86%, 85% of the spatial variation in nitric oxide, nitrogen dioxide, and nitrogen oxides concentrations, respectively.	Prenatal exposures to nitric oxide, nitrogen dioxide, and nitrogen oxides increased the risk of ALL and bilateral retinoblastoma in offspring. No associations were found for other cancer types or annual average exposures without temporal components
Vinceti et al., 2012	Northern Italy	1998–2009	0–14 years	83/332	leukeamias	benzene and PM_10_	sex, year of birth and province of residence during the diagnosis year.	Sex, age, province of residence, PM_10_, income and for other pollutants.	Benzene and PM_10_ exposures were estimated by modelling annual ambient air concentrations at geocoded residential addresses using ARC-GIS software. Benzene levels were estimated from traffic data and COPERT IV program.	Benzene exposure below the European Union limit of 5 m^3^/lg was associated with increased risk of leukemia in children under 5 years of age. A similar link for PM_10_ in this age group was attenuated after adjusting for benzene, suggesting it is not an independent risk factor.
Amigou et al., 2011	France	2003–2004	< 15 years	763/1,681	Acute leukemias	NO_2_	age and sex.	Parental education, housing type, urbanization birth order, early infections, preconception paternal smoking, maternal use of pesticides and residence next to a gas station.	Maternal interviews provided residence details. Traffic exposure indicators, including NO_2_ were estimated by integrating geocoded addresses, road network data, and national background air pollution data.	For both ALL and ANLL were linked to traffic exposure (road proximity or density and NO_2)_. The association is unlikely due to differences in disease severity or health care access for rural, semi-urban, and urban areas. Higher parental socioeconomic status was positively and significantly associated with the traffic indicators.
Brosselin et al., 2009	France	2003–2004	less than 15 years	765/1681	Acute leukemias	Benzene	Age and gender	Age, gender, number of children under 15 years of age, urbanisation level and housing type.	Maternal telephone interviews via Two databases (Catalist and Navteq) were used to validate petrol station proximity, with confirmation for 52% of controls and 54% of cases, indicating non-differential misclassification.	Residence next to petrol stations or automotive repair garages was significantly associated with acute leukemia, mainly in the intrauterine period.
Weng et al., 2009	Taiwan	1996–2006	0–14 years	729/729	Leukeamias	Traffic Exhaust as benzene and PAH	gender, year of birth, and year of death.	urbanization level.	Provincial Department of Health. PSD calculated using CPC/FPCC data, used as a proxy for municipality-level to estimate benzene exposure in a study of childhood leukemia mortality.	A significant exposure-response relationship between PSD and the risk of leukemia in young children after controlling possible confounders.

Moderate Resolution Imaging Spectroradiometer

Multi-angle Image SpectroRadiometer

Sea-viewing Wide Field-of-view Sensor

Normalized Difference Vegetation Index

Geographic Information System

Artificial Light At Night

Extremely Low Frequency -Magnetic Fields

Swiss National Cohort

Land-Use Regression

Polycyclic Organic Matter

Environmental Protection Agency

National-Scale Air Toxics Assessment

Swiss Childhood Cancer Registry

Polycyclic Aromatic Hydrocarbons

International Agency for Research on Cancer

Computer Programme to calculate Emissions from Road Transport

Petrol Station Density

Chinese Petroleum Corporation

Formosa Petrochemical Corporation

The most frequently investigated type of cancer was leukemia, reported in ten studies. Two studies focused exclusively on ALL, five assessed childhood cancer and three investigated two types of cancer, such as leukemia and lymphomas or leukemia and central nervous system (CNS) tumors. Additionally, one study assessed three cancer types (leukemia, lymphomas and CNS tumors), one focused on brain tumors and one assessed ALL, AML, non-Hodgkin lymphoma and CNS tumors.

The majority of the reviewed studies (five papers) focus on proximity to highways and traffic volume on the nearest highway as well as to bus or petrol stations and power lines. Three studies assessed the effect of benzene only; two investigated the effect of benzene and Nitric Dioxide (NO_2_); and another two focused on the effects of benzene, 1,3-butadiene and Polycyclic Organic Matter (POM). Two studies examined the effect of PM_2.5_, one was reviewed for benzene and PM_10_, one for PM_2.5_, PM_10_, NO_2_ and O_3_, one for benzene and Polycyclic Aromatic Hydrocarbons (PAHs), one for benzene, PAH and other air toxic, one for NO, NOx and NO_2_, one for PM_2.5_ and PM_10_, one for Ambient ultrafine particles (UFPs), one for PM_10_, NO_2,_ SO_2_ and lastly one for NO_2_.

### Challenges in the epidemiological studies

Table 2 below presents the methodological challenges, limitations and strengths of studies included in the scoping review. We discuss those challenges in five main aspects:

**Table 2:** Challenge in methodology, limitations and strengths of studies included in the scoping review

References	Challenge in methodology	Limitations	Strengths
Williams et al., 2024	The study linked cases and controls to the Yost index for area-level SES. Findings suggest a possible synergistic relationship between vegetation index and PM_2.5_ during early life on childhood cancer risk, possibly due to pesticide exposure. However, further research is needed due to limited sample size.	1-Hisorical PM_2.5_ and NDVI estimates for earlier births may cause exposure misclassification. 2-Residential mobility was not tracked, but its impact was likely minimal. 3-Total PM_2.5_ was considered without accounting for its chemical components. 4.The study was mainly urban, minimizing agricultural runoff concerns. 5-Individual SES was limited (maternal education data was missing for over 50% of participants). with need to use the area-level Yost index.	1-This large-scale study on early-life PM_2.5_ and NDVI exposures assumes minimal variation in pre-1998/2000 estimates, allowing to capture critical developmental windows beyond pregnancy. 2-This is one of the largest studies, making a significant contribution to the field.
Norzaee et al., 2024	Challenges like population mobility and changing addresses can bias study results. Small sample sizes and confounding factors are limitations. More research is needed to understand health impacts of magnetic fields from power lines. The exclusion of children who moved to Tehran for treatment after diagnosis may skew the study population.	1-Lack of individual level data on lifestyle and socio-economic data. 2-Use distance to pollution sources as highways as proxy for exposure, which does not account for factors like wind and geography. Despite this, the method still provides valuable exposure classification and does not invalidate the observed associations.	Not mentioned.
Zhong et al., 2023	Established risk factors for childhood ALL including Hispanic ethnicity, higher birth weight, lower birth order, caesarean delivery, and older parental ages.	1-Did not evaluate potential protective effects of green spaces. 2-Lack of comprehensive residential history, reducing study’s accuracy. 3-Could not differentiate between pollutant sources (pesticides vs. traffic-related). 4-Residual Confounding from unmeasured factors (e.g., smoking exposure) remains possible.	1-Large population-based design using linked California cancer and birth registry data. 2-High-quality data from comprehensive California Cancer Registry. 3-Minimized selection bias by avoiding direct participant contact. 4-Accurate exposure assessment using high-resolution ALAN and PM_2.5_ estimates from maternal addresses, reducing recall bias.
Malavolti et al., 2023	The study’s results should be interpreted with caution due to potential bias from unaccounted confounders (e.g., using paternal income as a proxy for household income, or medical ionizing radiation), statistical instability from a small sample size, and potential exposure misclassification from lack of residential stability or time- activity patterns (time spent at grandparents ‘home, school, or day care).	1-The study used 2017 fuel supply data for 2018–2019 due to data limitations. 2-Potential residual confounding and missing demographic and medical imaging information could affect results.	1-Avoided selection/information bias by not requiring active participation from participant contact lead to minimized bias. 2-Exposure assessment was blinded. 3-Sensitivity analysis showed similar results, indicating missing data was not a major confounder. 4-High residential stability was likely, reducing exposure misclassification.
Kreis et al., 2022	1- Advance exposure modeling by using LUR models to estimate NO_2_ and benzene levels 1991–2014. 2-Comprehensive data from the Swiss National Cohort, including demographic and socio-economic information (births, mortality, and migration) 3-Confounder control like socio-economic position, urbanization level, and background ionizing radiation.	1-Outcome and exposure misclassification from incomplete data and address histories. 2-Parially outdated covariates and less precise benzene estimates. 3-Likely non-differential misclassification, possibly attenuating hazard ratios. 4-Lacked data on lifestyle confounders such as nutrition or smoking.	1-Nationwide cohort with long-term precise residential data over 2.5 decades, minimizing selection bias. 2-Accurate, annual NO_2_ exposure models, captured exposure contrasts at a small spatial scale. 3-Accounted for key confounders like socio-demographics.
Lee et al., 2022	Residual Confounding: Possible due to unmeasured factors, despite adjusting for age, sex, and tobacco smoke). Data sources: used Korea’s NHIS database (2002–2012), detected 1,725 childhood cancer cases from a total of 1,261,855 children. Sample Size: Despite a large sample, the number of cancer cases was too low to precisely estimate risks for specific cancer types.	1-Unmeasured confounders would need to be very strong (E-value: 5.49), to fully explain the PM_2.5_ -cancer link. 2-Exposure misclassification from large area estimates and residential mobility, potentially shifting associations to the null. 3-Outcome misclassification from unverified diagnoses, also potentially shifting associations to the null. 4-Not accounted for prenatal exposure. 5-Limited generalizability to unique population genetic and PM composition.	The study used the National Health Insurance Service (NHIS) da se of Korea, a compulsory, nationwide claims database covering the entire population and provides its claims database from 2002. Its comprehensive, longitudinal nature makes it a good source for cohort studies.
Mazzei et al., 2022	The study used geocoded addresses to assess proximity to pollution sources and adjusted for several key confounders including NO_2_ levels, socio-economic position, highway distance, urbanization and years of existence of cantonal cancer registries. Repeated analyses were done for different time periods. Exposure may be misclassified as it was based on a single address rather than full residential history.	1-limited statistical power due to the small number of exposed cases. 2-Geocodes for petrol stations were only available intermittently from 1995 to 2011, causing potential misclassification errors. 3-Lack of full residential histories, leading to possible exposure misclassification. 4-Potential residual confounding from unobserved factors, including lack of data on reduced benzene emissions from petrol stations.	1-High-quality data by using the population-based Swiss Childhood Cancer Registry (SCCR), (95% completeness) with precise geocodes (93% within 50 m). 2-No participants were lost due to missing data, except for missing geocodes. 3-Study Focus: The study assessed the risks of childhood central nervous system (CNS) tumors in relation to the proximity of residences to petrol stations. 4-Minimized selection bias and differential misclassification. controls were sampled from national censuses. 5-Accuracy: Exposure was assessed based on exact geocodes of children’s residences and petrol stations, minimizing misclassification bias. 6-Comprehensive adjustment for confounders: like background levels of air pollution and proximity to highways.
Lavigne et al., 2020	1-Exposure misclassification UFP estimates based on short-term monitoring data (2010–2011), introducing temporal errors that likely biased results toward the null. 2-Residual Confounding: due to the lack of individual-level data on key confounders (income, education, ethnicity, indoor air pollution, passive smoking, and magnetic field exposure), potentially lead to adjusted bias. These challenges emphasize the need for more precise exposure assessment and comprehensive confounder data in future studies.	1-Pollutant correlation: UFPs may correlate with other air pollutants, such as benzene, volatile organic compounds (VOCs), and remain potential confounders. 2- Residential Mobility: imprecise data on moving date, leading to potential exposure misclassification. 3- Rare outcomes: the rarity of pediatric cancers, limited analysis of specific subtypes and precise risk estimates.	1-Spatiotemporal- resolved air pollution estimates. 2-large, population-based sample minimizing selection bias. 3-adjustment for key confounders using a province-wide cancer registry. 4-First study to investigate UFPs exposure and childhood cancer risk.
Peckham-Gregory et al., 2019	Area-level poverty, from the US Census, ≥ 15% defined as high poverty areas, was used as the SES measure. Potential for residual confounding by unmeasured factors like race/ethnicity, education, and SES. Exposure misclassification from residential mobility and inability to assess short-distance exposure Lack of data on other maternal health behaviors and environmental risk factors.	1-Using maternal proximity to major roadways as a proxy for air pollution exposure, given the limitations of direct monitoring. 2-Exposure misclassification possible from residential mobility, while local move may decrease this bias. 3-Focus on Urban areas and a 500-meter limit may not capture all variables or shorter exposure distances. 4-Using birth records and geocoding data may delete key variables.	This large, population-based Texas study (16 years, > 2000 cases) found a consistent link between maternal proximity to major roadways and childhood leukemia risk, highlighting the reliability of birth certificates as data sources.
Seifi et al., 2019	The study faced a temporal mismatch between exposure (2010 LUR model) and diagnosis years (2007–2009). Using randomly generated controls instead of real population. Lacked data on key confounders as SES and parental factors.	1-Analysis was unadjusted, potential residual confounder risk. 2-Exposure misclassification. 3-Possible selection bias. 4-Small sample limited statistical power and cancer subtype analysis.	1-First study in Iran on air pollution and childhood cancer, used a validated GIS-based LUR model with high spatial resolution. 2-Based on population-based registry. 3- Comprehensive urban exposure coverage. 4-Provided valuable evidence linking PM_10_ exposure to childhood cancer.
Raaschou-Nielsen et al., 2018	Confounders were selected a priori based on their potential association with benzene levels and childhood cancer risk. 2-Analystical approach was to follow the prior research for consistency. 3-Sensitivity analyses to test the robustness of results and enhance comparability by: a-Exposure metrics (quartile vs. percentiles). b-Exposure calculation (time-weighted averages (TWA) vs. cumulative exposure). c- Residential data (single address vs. full history).	1-Nondifferential Misclassification: model-based exposure assessment likely biased the relative risk estimate towards 1.0. 2-Exposure Group Limitations: limited number of cases in the highest exposure group. 3-Lack of data on key confounders as maternal occupational exposure to benzene.	1-Using reliable national registries (Danish Cancer population) to identify cases and controls. 2-Access to complete residential histories. 3-applied a validated exposure model to a large dataset
von Ehrenstein et al., 2016	1-Residual Confounding: possible despite adjustment for key confounders (parity, child sex, SES indicators, and rural/urban location) and sensitivity analyses. 2-Limited Power: due to the rarity of childhood brain tumors and limited air toxics monitoring data, despite the large overall sample. 3-Field-Specific Limitation: Small subtype numbers are a common challenge in this research area.	1-Unmeasured exposures: lack of data on indoor air, occupation, smoking, and diet. 2-Residential mobility: exposure misclassification from using birth addresses, though most moves are local (< 10 km). 3-Incomplete early-life data: no address history for the first year of life. 4-Control group differences: demographic differences between cases and controls may affect exposure estimates.	This population-based, record-based study minimized recall bias and selection bias while enabling detailed analysis of brain cancer subtypes.
Magnani et al., 2016	1-Assessed traffic Intensity: using road type and length. 2-Adjustment for key confounders (parental exposure to solvents, education, and smoking). 3- Modeled outdoor benzene with high accuracy. 4-Measured ELF-EMF exposure in homes via interviews to reduce recall bias. 5-Several sensitivity analyses were conducted to test exposure (at different life stages, focusing on the house at birth, the most exposed house, and homes), showed consistent non-No significant results.	1-Potential recall and participation bias though associations appeared specific for AnLL and AML. 2-Limited statistical power for specific leukemia types, especially AnLL and AML. 3-Findings suggest recall bias was not a major factor, highlighting the need for leukemia type specific analyses in future studies.	1-SETIL Study: Large, population-based case-control study, with complete disease and age coverage. 2-Case Confirmation: Cases confirmed at the central Italian Association of Paediatric Haematology and Oncology (AIEOP) laboratory; age range 0–10 chosen for better parental knowledge of exposures. 3-High participation percentage (nearly complete for cases; 70.8% for controls, comparable to other studies.
Symanski et al., 2016	1-Potential overestimation of historical pollutant levels (1996 POM and 2002 benzene levels). 2-Limited temporal data from NATA data were in (1996, 1999, 2002 and 2005), possibly introducing errors. 3-No residential history data during pregnancy or after birth. 4-Limited power for subtype or age specific analysis.	The study used U.S. EPA NATA modelled data as a proxy for personal exposure, which is affected by air pollutant levels in various outdoor and indoor settings and time spent in each microenvironment.	1-Texas Cancer Registry’s extensive data enabled a large population-based study on early ALL. 2- Texas had extensive road traffic in and around large urban areas and a large number of petrochemical plants. 3-Using robust registry data to minimize selection bias. 4-Included maternal smoking data and confirmed robustness with sensitivity analysis.
Spycher et al., 2015	1-The study considered various confounding factors such as urbanization, socio-economic position, education level, crowding, nationality, background radiation, and proximity to power lines and EMF sources. 2-To address exposure misclassification due to mobility, analyses were repeated for children with stable residences.	1-Used proximity to highways as a crude measure of traffic pollution, lacked data on other roads or background pollution, complete address histories. 2-Traffic volume data was only from 2005, causing misclassification in exposures and outcomes. 3-Potential for exposure and outcome misclassification due to geocoding errors, record linkage issues and incomplete cancer registration.	1-Used high-quality national registries (SNC/SCCR), with 95% case coverage. 2-Individual-level data minimized selection or response biases. 3-Cohort design provided greater statistical power and accounted for exposure changes over time due to new highway construction and mobility.
Houot et al., 2015	1-Results were robust (unchanged after excluding Down’s syndrome). 2-Unlike to be confounded by other known risk factors. Since the potential factors should be specific to AML and more prevalent in places with a higher major road density. 3-Traffic-related benzene is likely the main exposure. 4-Nitrogen dioxide concentration was derived from a large grid, leading to potential non differential misclassification than the distance or road length metrics individually calculated from vectorized maps.	1-Lacked complete residential histories, 2-Lacked individual data on potential childhood leukemia risk factors, except for age. 3- The study didn’t account for household benzene sources besides traffic. 4- potential for exposure misclassification if critical exposure windows of differential moving patterns were missed.	1-The GEOCAP Study used recent historical databases to avoid selection by differential participation and misclassification, and recall bias. 2-Cases identified through a comprehensive national registry, and controls were randomly sampled to prevent selection bias. 3-Addresses were geocoded, and road metrics were calculated objectively. 4-enhance exposure assessment by combining benzene estimates and road length.
Heck et al., 2014	1-Used only birth addresses, which may cause exposure misclassification, especially for the 1 st trimester. 2-Monitors were up to 6 km from the home, may cause exposure misclassification.	1-Lacked data on key exposures (parental smoking, individual PAH). 2-Results should be interpreted cautiously due to multiple statistical tests. 3-Observed associations may reflect benzene’s known leukemogenic effect.	1-First study to link childhood leukemia with PAH exposure. 2-used population-based data, avoiding biases from responses or recall. 3-Findings consistent with previous research on cancer risk from traffic pollution (IARC, 1989).
Badaloni et al., 2013	1-Estimated traffic exposure using road proximity and intensity. 2-. Unconditional regression was chosen due to missing corresponding controls or cases. 3-Potential bias might exist in case and control selection, although the age and gender distribution of cases matches population cancer registry data.	1-Pre-diagnosis exposure models and road network data. 2- The PM_2.5_ model’s 4 km × 4 km resolution indicates background exposure levels. 3–10% of the population was excluded due to missing data. 4-Time-dependent exposure and preconception exposure could not be assessed.	1-First national Italian study on childhood leukemia and air pollution, using national registries with over 95% coverage. 2- Minimized exposure misclassification by restricting to stable residences. 3-Used two traffic indicators, several pollutants, and novel LUR models for fine grid estimates.
Ghosh et al., 2013	1-Controls were randomly selected from birth records (20:1 ratio) and matched by birth year. 2-They did not have sufficient information to assign childhood air pollution exposures. 3-Not all monitors were active for the entire study period. 4-incomplete air pollution data lead to some exclusions. 5- Adjustment for multiple socioeconomic and perinatal factors, but these did not change the results for ALL. 6- Despite potential selection bias, results were consistent across datasets. 7- Birth certificate addresses were used as proxies for residential locations, potentially causing misclassification. 8- The potential bias from controls moving out of state is minimal due to the rarity of these diseases.	1-Uses birth certificate addresses reflect pregnancy exposures, despite 20% of women moving during pregnancy, but pollution changes were likely minor. 2-No data on early childhood air pollution exposures. 3-Potential residual confounding (smoking and parental occupations). 4- The LUR model measured traffic markers as nitric oxide, nitrogen dioxide, and nitrogen oxides, not specific causal air toxins.	1-Using spatiotemporal LUR model in childhood cancer research. 2-Improved exposure accuracy by integrating spatial predictors with temporal monitoring data. 3-Enabled precise analysis of specific pregnancy periods. 4-Methods validated by sensitive analyses showing consistent results. 5- Using birth certificate linkage, it captured many childhood cancer cases and minimized participation bias.
Vinceti et al., 2012	1-Socioeconomic factors, magnetic field exposure, and PM_10_ from traffic did not explain the findings. 2- Higher relative risks (RRs) for benzene exposure were observed in children under 5, likely due to less exposure misclassification and more time spent at home. 3-The exact exposure amount linked to increased leukemia risk is unclear due to unknown induction periods. 4- Results may also reflect confounding from unmeasured pollutants or other factors.	1-Lacked direct subject data on potential confounders, though key variables (income, genetics) were addressed. 2-Known risk factors for childhood leukemia are inconsistent, except for radiation and genetic susceptibility. 3- Despite adjustment, unmeasured pollutants And factors could still influence results. 4-Used estimated ambient air levels with potential errors, limited validation. 5-Measurement errors likely biased risk estimates towards the null. 6-Focused on benzene and PM_10_, unable to fully separate their effects from other correlated pollutants. 7-Using residential location at diagnosis might have caused exposure misclassification. 8- Cases in mountainous areas were excluded due to modelling limitations.	1-The study analyzed age-related susceptibilities and leukemia subtypes, noting that PM_10_ exposure was underestimated. 2-PM_10_ was evaluated as a potential confounder and independent risk factor for leukemia, with a weaker but possibly elevated association compared to benzene.
Amigou et al., 2011	1-Proximity to heavy-traffic roads was measured within 500 m of residences, with categories based on road class. 2- Manual address location was used when needed, but 2.4% of subjects were only located by municipality using Native Road data. 3-Using only the last address, potentially causing misclassification if relevant exposure was prenatal or early childhood.	1-Assumed 2000 NO_2_ levels reflected later periods (2003–2004), likely attenuating true associations. 2-Using only the last address, potentially causing misclassification if relevant exposure was prenatal or early childhood.	1-Cases were identified by the French National Registry, minimising selection bias. 2-Controls selected via random-digit dialing, preventing socioeconomic selection bias. 3-Exposure data from residential addresses avoided recall bias. 4-Socioeconomic and residence type confounders were adjusted for and stratified.
Brosselin et al., 2009	1-Recall bias was noted, with case mothers reporting exposure more often than control mothers. 2-The proximity of petrol stations was confirmed for similar percentages of controls (52%) and cases (54%), indicating non-differential misclassification.	1-The questionnaire did not account for business closures while children lived at a residence, and many petrol stations closed in the 1990s. 2-The study had enough statistical power for many associations but not for period, duration of exposure, or subtype analyses.	1-Cases identified through the comprehensive French National Registry, covering 99% of acute leukemia cases in mainland France. 2-Many potential confounders, such as urban/rural status, type of housing, parental smoking, and pesticide use, were considered.
Weng et al., 2009	1-Used an urbanisation index as a proxy for SES due to unknown individual SES. 2-This index adjusted for confounding from different urbanisation levels. 3- Minimized misdiagnosis through verified causes of death data. 4- Based on the “usual place of residence” for children aged 0–14), minimising occupational exposure confounding. 4- Migration due to leukemia diagnosis could introduce bias.	1- Non-differential misclassification likely from unmeasured individual activities and indoor sources, migration, biased association magnitude. 2- Key confounders for leukemia, like radiation, parental occupations, and genetic disorders, were not included in the study.	1-Taiwan’s death registration system is accurate due to mandatory registration and annual verification. 2-Equal access to medical care, minimized regional outcome bias. 3- Migration due to diagnosis was unlikely, as parental job changes were minimised and urbanisation level was controlled

#### Recall and selection biases

Most studies included in this review were case-control studies, and therefore were at risk of recall and selection biases. We classified the studies by the types of bias they identified, based on how the original authors described their limitations and also applied predefined criteria based on standard epidemiological definitions.

Studies were classified as being subject to recall bias (in case-control studies) when participants were systematically more (cases) or less (controls) likely to remember and report information about exposure based on their outcome status, or to recall details about their outcome depending on their exposure, through a questionnaire or interviews.

Selection bias, on the other hand, arises when the study participants do not accurately reflect the study population (through hospital registries or incomplete datasets). This may occur because of the improper specification of inclusion criteria or an incorrect selection of the population (even when inclusion criteria are well defined).

Conversely, studies were considered to have minimized bias if they relied on objective data sources, such as registries and census data [46, 47]. We used this approach to ensure a consistent and transparent classification of bias across studies.

##### Studies with likely selection bias

Vinceti et al. (2012) identified cases from a hospital-based registry and controls from population data (National Health Services Local Health Units of Modena and Reggio Emilia) observing potential selection and recall biases due to their methodology [48]. Similarly, Badaloni et al. (2013) identified cases from a national registry of childhood neoplasms and recorded them at diagnosis using a common registration method including patient’s personal identification data, while controls were randomly sampled from population registries. The authors noted potential selection bias in case and control selection [49]. In Seifi et al. (2019), selection bias was possible due to the use of randomly generated GIS points (non-individual controls) and the exclusion of real population data. This approach meant that controls may not have shared the same selection probabilities as cases [50].

##### Studies with likely recall bias

Brosselin et al. (2009) utilized standardized telephone interviews with mothers to validate addresses and reported recall bias, with case mothers more frequently reporting exposure than control mothers across all age groups (9.3% for children aged 4 years; 9.1% for children aged over 11 years). This discrepancy in reporting suggests that case mothers might have been more likely to recall and report exposure due to their child’s illness, rather than there being a true association between the exposure and the development of leukemia [51]. Magnani et al. (2016) employed questionnaire interviews where the parents of cases and controls were interviewed at home with a structured questionnaire administered by trained interviewers, highlighting potential recall and participation biases [52].

##### Studies that minimized potential biases

In contrast, some studies effectively minimized potential biases.

For example, Amigou et al. (2011) interviewed mothers of case and control children, gathering data on residential characteristics such as name of the municipality and its postal code, housing type (apartment, house, or farm) and nearby businesses potentially emitting harmful pollutants, such as petrol stations and automotive repair garages. The risk of recall bias was minimal because exposure was derived from residential addresses, not self-reported data. Similarly, selection bias was limited because controls were identified through randomly unlisted telephone numbers. Researchers randomly selected 60,000 phone numbers representative of the French population from the national directory and then incremented each number by one to include unlisted numbers, maintaining similar geographic and demographic distributions [53].

Moreover, Heck et al. (2014) collected data on airborne toxic pollutants over an 18-year period (1990–2007), thereby avoiding response and recall biases by using existing records for data linkage. This long-term and record-based design enhanced the reliability and comprehensiveness of findings, enabling robust analysis of long-term effects of exposure to ambient air toxics childhood leukemia risk [54].

Similarly, von Ehrenstein et al. (2016), carried out a population-based design and record-based approach to eliminate biases. This method avoids recall bias because it relies on existing records rather than participants’ memories. It also reduces selection bias by including all eligible cases and controls from population records, ensuring that nonparticipation does not skew the results. Additionally, this approach allows for the differentiation between brain cancer subtypes, providing more precise and reliable data [55].

Zhong et al. (2023) minimised selection and recall biases by using maternal residential address at the time of delivery, documented in birth records for exposure estimates prior to the diagnosis of childhood ALL, thus avoiding direct contact with participants [56]. Spycher et al. (2015) used individual data from the entire childhood population of Switzerland based on the 1990 and 2000 national censuses. Given all data were sourced from routine and high-coverage records, the study is likely devoid of selection or response bias that might have influenced earlier case-control studies [57].

Kreis et al. (2022) relied entirely on population-based routine datasets, allowing for a relatively large sample size compared to previous studies on childhood cancer and traffic-related air pollution. Specifically, they included 2,960 cancer cases identified from the Swiss Childhood Cancer Registry, which is substantial for this type of research and helps minimize the risk of selection bias [58]. Furthermore, Malavolti et al. (2023) conducted an exposure assessment using geocoded addresses of child residences and petrol stations. This method allowed them to estimate the distance from each child’s residence to the nearest petrol station without needing active involvement from participants, their families, or petrol station staff. By using this approach, they avoided potential selection and information bias (which can occur if participants provide inaccurate information) [59].

In addition, Houot et al. (2015) mitigated selection and recall biases in their study by using historical databases and geocoded addresses, allowing them to determine residential proximity to heavy-traffic roads without relying on participant recall or active involvement. By employing a large sample size of 2,760 cases and 30,000 controls, they ensured robust and reliable results, free from contemporary influences or self-reporting inaccuracies [60]. Similarly, in Symanski et al. (2016), selection bias was reduced by using birth records to identify controls that accurately represented the source population, as they were matched by birth month and year to the cases. This approach ensured proper matching to the cases, minimizing bias [61]. In Seifi et al. (2019), recall bias was unlikely because they used exposure data from GIS-based LUR models, and cases extracted from a registry, no interviews or questionnaires [50].

#### Air pollution assessment

Exposure to outdoor air pollution consists of a mixture of traffic combustion by-products, heating and industrial emissions. These pollutants can be assessed either collectively as a mixture or individually as distinct particles [4]. Several studies have used a variety of methodological approaches, each with strengths and limitations.

##### Proximity-based measures

Several studies used residential proximity to pollution sources as a proxy for exposure. For example, Brosselin et al. (2009) found non-differential exposure misclassification in their study on the proximity of petrol stations to residences. This misclassification occurred because the percentages of proximity confirmation were similar for both controls (52%) and cases (54%). However, the extent of this misclassification could not be precisely determined due to the lack of comprehensive gold-standard sources [51]. Peckham-Gregory et al. (2019) used geographic information system (GIS) to assess residential proximity to major roadways, noting limitations such as inconsistent air monitor placement and potential misclassification due to residential mobility [62]. Other studies used petrol station density. Mazzei et al. (2022) calculated distances to petrol stations but did not account for entire residential histories, leading to potential misclassification [63]. Weng et al. (2009) used petrol station density as an indicator of benzene and hydrocarbon exposure, highlighting potential misclassification due to variations in traffic patterns and other environmental factors. Migration between areas of different petrol station densities could have introduced bias, but the study population’s low mobility likely minimized this effect. Any exposure misclassification was likely nondifferential, reducing the estimated association rather than introducing a positive bias [64].

Other studies focused on distance-based indicators. Amigou et al. (2011) assessed NO_2_ exposure from traffic emissions, with limitations in data accuracy over time [53]. Houot et al. (2015) estimated exposure to nitrogen dioxide and benzene based on proximity to major roads, facing potential misclassification due to arise from using addresses at the time of diagnosis and inclusion, without considering complete residential history, which might have led to inaccurate cumulative exposure assessments, individual childhood leukaemia risk factors, or household sources of benzene exposure [60]. Magnani et al. (2016) predicted benzene concentrations near residences but struggled with detailed location descriptions because the information collected through questionnaires and geocoding of home addresses was not precise enough. This imprecision could lead to challenges in accurately determining the exact locations where the children lived, and therefore, estimating their exposure levels to traffic pollution more accurately [52]. Spycher et al. (2015) assessed highway proximity and traffic volume, with limitations in address histories and data accuracy [57]. Norzaee et al. (2024) used a GIS-based approach with circular buffers around various infrastructure to evaluate land use exposure, using distance as a pollution proxy, but noted limitations in not assessing specific toxins and the influence of environmental factors like wind and geography [65].

##### Land-use regression (LUR) and dispersion models

More advanced modelling techniques, such as LUR and dispersion models, were used to estimate PM, NO_2_ and benzene concentrations. Badaloni et al. (2013) assessed PM_2.5_, PM_10_, NO_2_, and O_3_ exposure using GIS, traffic indicators, a dispersion model, and LUR models with satellite data. Limitations included outdated road network data, low PM_2.5_ model resolution, missing exposure or confounding variables for 10% of the population, and inability to account for residential history or preconception exposure, leading to potential misclassification [49]. Ghosh et al. (2013) used LUR to assess nitrogen oxides variability in Los Angeles but encountered limitations such as reliance on birth certificate addresses, potential residential mobility, and insufficient network density for validating seasonalized LUR estimates [10]. Similarly, Seifi et al. (2019) used the LUR model to estimate annual PM_10_, NO_2_, and SO_2_ concentrations at children’ residential addresses in Tehran. They relied on 2010 data to represent exposures during 2007–2009, with no personal or temporal information, introducing potential exposure misclassification [50].

Kreis et al. (2022) used dispersion models and LUR for NO_2_ at children’s residences, with issues in spatial covariates and benzene monitoring [58]. Raaschou-Nielsen et al. (2018) calculated benzene exposure using the Operational Street Pollution Model, with potential bias from misclassification. The study faced limitations such as spatial and temporal variability and difficulty isolating benzene exposure from other pollutants and lifestyle factors. These studies highlight the need for accurate, up-to-date data in air pollution assessment [66]. Lavigne et al. (2020) estimated ultrafine particles (UFP) with mobile monitoring data and adjusted NO_2_ exposure weekly, focusing on less-studied UFPs and prenatal exposure periods with robust statistical analysis. However, limitations included difficulty in accurately measuring UFPs, potential exposure misclassification due to residential mobility, and reliance on short-term data [67].

##### Registry and modeled exposure data

Several studies relied on modelled or registry data linked to residential addresses. Symanski et al. (2016) linked maternal addresses to U.S. Environmental Protection Agency (EPA) data to model benzene and other pollutants, noted overestimation in 1996 POM levels and 2002 benzene levels. Data limitations included reliance on U.S. EPA National-Scale Air Toxics Assessment (NATA) modelled data as a proxy for personal exposure, lack of detailed air pollutant measurements for many census tracts, limited NATA data availability for certain years, insufficient data on maternal mobility during pregnancy, and constraints on examining disease risks by subtype or age at diagnosis due to limited power [61]. von Ehrenstein et al. (2016) assessed exposure to 42 carcinogens using geocoded addresses and air toxics data from 31 monitors in California. The study’s limitations included reliance on The California Air Resources Board (CARB) data, which only covered areas within a 5-mile radius of the monitors, potentially leading to misclassification for those living farther away, and the use of geocoded addresses and ZIP code centroids, which might not accurately reflect individual exposure levels [55]. Similarly, Vinceti et al. (2012) assessed benzene and PM10 exposure using geocoded residential addresses and traffic emission models, facing potential errors and limited validation due to reliance on geocoded residential addresses and traffic emission models, which may not accurately capture long-term exposure levels and could lead to misclassification of exposure [48]. Zhong et al. (2023) estimated daily PM_2.5_ levels but could not evaluate PM composition due to limitations in their measurement methods, which focused on overall particulate matter concentration rather than its specific components [56]. Williams et al. (2024) used a global model combining NASA datasets to assign yearly average PM_2.5_ values based on birth year, capturing early-life exposures but facing limitations with pre-1998 data and residential history [68].

##### Fixed-site monitoring and national air quality networks

Other studies linked outcomes to stationary monitoring data. Heck et al. (2014) studied air toxics exposure using community monitors at 39 sites, analysing 42 carcinogens, but faced limitations such as reliance on birth addresses, potential misclassification from monitor distance, and inability to assess individual Polycyclic Aromatic Hydrocarbons (PAH) exposure [54]. Likewise, Lee et al. (2022) used AirKorea data to assess monthly mean concentrations of key air pollutants across various regions in Korea, employing a detailed air quality model for PM_2.5_ levels before 2015 and accounting for residential changes, leading to a more precise exposure assessment and stronger statistical associations. The study collected monthly mean concentrations of key air pollutants (PM_10_, PM_2.5_, NO_2_, SO_2_, CO, and O_3_) from AirKorea’s monitoring stations. PM_10_ and PM_2.5_ were measured using the β-ray absorption method, and O_3_ was measured using the ultraviolet photometric method. For PM_2.5_ data before 2015, the study used modelled estimates based on meteorological data and emissions inventories. Children’s residential addresses were tracked to assign these pollutant concentrations for exposure analysis. The study faced limitations such as potential measurement errors due to reliance on historical air pollution data, possible misclassification of exposure levels, and challenges in accounting for individual residential mobility [69].

#### The impact of different periods of air pollution exposure on the risk of childhood cancer

Several studies have examined the link between outdoor air pollution and childhood cancer, focusing on exposure during pregnancy and early childhood. Badaloni et al. (2013) examined traffic-related pollutants during conception, birth, and diagnosis. The study found no significant association, indicating that results do not vary based on the time periods of exposure assessment [49]. von Ehrenstein et al. (2016) examined whether exposure to air toxics during pregnancy and early life is associated with an increased risk of childhood brain tumors. The study found that prenatal and early-life exposure to certain air toxics was linked to a higher risk of specific brain tumors, such as primitive neuroectodermal tumors (PNET) and medulloblastoma, but not all types of brain tumor [55]. Magnani et al. (2016) conducted sensitivity analyses on exposure during different life periods, including the address at birth and the address with the highest exposure. They found that exposure to road traffic pollution increases the risk of certain leukemias, particularly Acute Non-Lymphoblastic Leukemia (ANLL). The risk varies depending on the time periods and levels of exposure assessed [52]. Lavigne et al. (2020) tracked ultrafine particle exposure weekly during pregnancy and monthly during childhood, finding a link to higher cancer incidence before age six. The results suggest that different time periods of exposure can affect the incidence of childhood cancers [67].

Additionally, Heck et al. (2014) investigated the risk of leukemia in relation to exposure to ambient air toxics during pregnancy and early childhood. They found that third-trimester exposure to certain toxics increased leukemia risk, particularly ALL and AML [54]. Peckham-Gregory et al. (2019) examined whether living near major roadways affected the risk of childhood leukemia. The study found no strong association between maternal proximity to major roadways and childhood acute leukemia (ALL or AML). The impact of different time periods was not analyzed [62]. Ghosh et al. (2013) found that prenatal exposure to traffic-related air pollution increased the risk of ALL and bilateral retinoblastoma, especially during the first and second trimesters. The study specifically analyzed all three trimesters to identify these susceptible windows of exposure [10]. Despite limitations, these studies highlight associations between pollution exposure and increased risks of childhood cancers, emphasizing the critical periods of pregnancy and early childhood for assessing these risks. These periods are particularly sensitive due to the developing body’s increased vulnerability to environmental stressors.

#### Data sources for cases

In studies that investigate the association between the exposure to outdoor air pollution and childhood cancer risk, a valid diagnosis of the disease is essential. In health services research, claims data and cancer registry data play as an important tool for identifying cases.

Several studies have used regional or national cancer registries to collect comprehensive data, such as studies in France, Italy, Texas (USA), Switzerland, California (USA), and Denmark. In these countries the cancer registries provide extensive coverage of childhood leukemia and other cancers. This data is crucial for large-scale and reliable cancer research, minimizing bias and ensuring robust sample sizes [10, 49, 51–57, 60, 62, 66, 68].

Other studies have used childhood cancer registries to gather comprehensive data. Mazzei et al. (2022) and Kreis et al. (2022) utilized the Swiss Childhood Cancer Registry, while Lavigne et al. (2020) linked mother-infant pairs with cancer cases in Ontario from the Paediatric Oncology Group of Ontario Networked Information System (POGONIS) [58, 63, 67]. Additionally, Seifi et al. (2019) identified childhood cancer cases in Tehran from the Center for the Control of Non-Communicable Disease, a population-based registry managed by the Iranian Ministry of Health [50]. These studies highlight the value of comprehensive, population-based registries in providing reliable data for research. Alternatively, some studies used hospital registries for data collection, like Norzaee et al. (2024) that conducted a case-control study on leukemia and lymphoma in Tehran using data from three hospitals [65]. Vinceti et al. (2012) and Malavolti et al. (2023) conducted similar studies in Italy, using hospital registries to identify cases. These studies ensured socioeconomic similarity and reliable data by matching cases to various factors [48, 59]. Although, the advancements in medical technology have made cancer diagnoses highly accurate, with a small risk of misclassification.

Furthermore, Lee et al. (2022) used data from Korea’s National Health Insurance Service (NHIS) for a cohort study, providing comprehensive data ideal for longitudinal studies due to its extensive and continuous data collection [69]. Differently, Weng et al. (2009) analyzed childhood leukemia deaths in Taiwan using accurate data from the Bureau of Vital Statistics, ensuring reliable results and consistent medical care access across regions [64].

#### Data sources for controls

Several studies have sourced controls from a variety of sources. This selection is crucial because it directly affects the study’s validity and reliability by minimizing bias, enhancing comparability, reducing confounding, ensuring representativeness, and improving statistical power.

##### Population-based controls

Badaloni et al. (2013) randomly selected controls from population registries, matched by birth date, gender, and region. They conducted face-to-face interviews with parents. The method ensured comparability through random selection and high participation rates but faced potential bias from non-blind interviews and a percentage of 69% of family refusal for controls [49]. Rasschou-Nielsen et al. (2018) selected controls from the Danish registry by sex, age, and calendar time. Each case had two to five controls for each type of the studied cancer [66]. Houot et al. (2015) selected controls randomly from the French population under 15 years, stratified by area. Controls (*n* = 30,000) were representative demographically and socioeconomically. This prevented selection bias but lacked complete residential history and not accounting for individual risk factors [60]. von Ehrenstein et al. (2016) randomly selected controls from California birth rolls, matched 20:1 by birth year to childhood cancer cases. This study has a large sample size (n = = 30,569) minimised recall and selection bias but faced residual confounding, limited geocoding accuracy, and reduced power for rare tumors [55]. Similarly, Seifi et al. (2019) randomly selected population-based controls within Tehran using GIS, unrelated to exposure status, and applied 4:1 control to case ratio, and this reduced interviewer bias [50].

##### Birth record-based controls

Symanski et al. (2016) sampled controls from Texas Department of State Health Services (DSHS) birth records (1991–2009) with a 10:1 matching ratio for cases. Excluded records included those with missing geocoding data, non-singleton births, and birth defects. Using birth records minimized selection bias but potentially affected comprehensiveness due to exclusions [61]. Similarly, Peckham-Gregory et al. (2019) selected controls from Texas birth records (1995–2011) with matching (1:10 ratio) by birth year, excluding entries in the Texas Cancer Registry. This selection provides strong power to the study [62]. Zhong et al. (2023) randomly selected 50 controls per ALL case from birth records, based on birth year. By using pre-diagnosis maternal addresses lead to minimizing selection and recall bias [56]. Likewise, Williams et al. (2024) used birth records to identify controls and matched them to cases by sex and birth year (12:1 ratio), this method of selection ensures population representation, though lacking post-birth residential history could lead to exposure misclassification [68]. Similarly, Ghosh et al. (2013) randomly selected controls from birth records of children without cancer (20:1 ratio) and matched by birth year. This provided a strong comparison group (80,658 controls), but faced potential biases from residential mobility and missing/excluded data [10]. Additionally, Heck et al. (2014) selected controls randomly from California birth certificates matched by birth years with cases (20:1 ratio). This selection provided a large control group, but faced exclusions and potential geocoding inaccuracies before 1998 [54].

##### Hospital-based controls

Norzaee et al. (2024) used a hospital-based case-control approach, randomly selecting controls from the same hospitals as cases, matched by socioeconomic criteria, age, and residency (1:1) [65]. Vinceti et al. (2012) selected controls randomly to minimize bias from population data recorded by local health units, with matching of (1 case:4 controls ratio) by sex, birth year, and province of residence during the diagnosis year [48].

##### Telephone and directory-based controls

Brosselin et al. (2009) matched cases by age and gender using quota sampling with a 1:2 ratio and conducted structured interviews with mothers. This method reduced selection bias and enhanced comparability but faced representativeness issues and bias due to excluding cell phone-only users [51]. Amigou et al. (2011) used quota-sampling from 60,000 phone numbers in the French population directory, ensuring regional and urban representation. Quotas matched controls age and sex to cases (1 case:2 controls ratio). Of 50,217 numbers dialed, 1,681 mothers participated in the interviews. This reduced selection bias but faced potential non-participation and household exclusion biases [53].

##### National health service or census controls

Mazzei et al. (2022) and Kreis et al. (2022) sampled controls from the Swiss National Cohort, matched by age, sex, and census year (1case:10 controls ratio). This method ensured high statistical power and minimized selection bias, but faced potential biases from sampling between censuses [58, 63]. Malavolti et al. (2023) matched four controls per case by sex, birth year, and province from the National Health Service directory. This method avoided bias but faced incomplete residential history and unmeasured confounding [59]. Magnani et al. (2016) randomly sampled controls from National health Service rosters, matched by birth date (± 15 days), gender, and region (1 case:2 controls ratio). Home interviews were conducted using structured questionnaires. This study achieved a good participation percentage (70.8%) and used a representative design. However, there were potential risks of recall and participation biases [52].

##### Mortality base-controls

Weng et al. (2009) matched controls to cancer cases by gender, birth year, and death year (1:1 ratio), excluding deaths from neoplasms and respiratory causes. This method assessed traffic air pollution’s impact on leukemia mortally [64].

#### Confounders and effect modifiers

Several studies investigate the possibility of confounding and effect modification that may arise while studying the association between air pollution exposures and the risk of childhood cancer. However, Seifi et al. (2019) did not account for any confounding factors, reporting only unadjusted odds ratios for PM_10_, NO_2_, and SO_2_. Subsequently, lead to introduce residual confounding [50].

##### Socioeconomic factors and urbanization

Amigou et al. (2011) found that the results remained unchanged regardless of adjustments for socioeconomic factors, type of residence, parental education, and urbanization. This indicates that the outcomes did not vary, whether or not these variables were controlled for [53]. Spycher et al. (2015) adjusted for sex, birth year, socioeconomic factors, ionizing radiation, and electromagnetic fields and found that the results remained largely unchanged. The study consistently indicated an association between living close to highways and an increased risk of childhood cancer, regardless of the analytical method used [57]. Peckham-Gregory et al. (2019) used area-level poverty as a proxy for socioeconomic status, adjusting for race/ethnicity, education, and socioeconomic status (SES), noting urban exposure levels but limited by birth record data. After adjusting for various factors, the results showed little change, indicating that living close to major roadways did not significantly affect the risk of childhood leukemia [62].

Von Ehrenstein et al. (2016) adjusted for birth year, maternal age, race/ethnicity, birthplace, education, and other factors. This adjustment did not significantly change the results, suggesting that future studies should address residual confounding to confirm associations with childhood brain cancer [55]. Mazzei et al. (2022) adjusted for NO₂ levels, traffic pollution, socio-economic position, and urbanization, found that after adjusting for these factors, the odds ratio for cancer diagnosis increased from 1.29 to 1.77, indicating a stronger association between living near petrol stations and childhood cancer [63].

Moreover, Weng et al. (2009) used urbanization as a proxy for SES, showing a link between particulate matter and leukemia risk [64]. Norzaee et al. (2024) adjusted for age, sex, parental smoking, and family history, finding significant associations between proximity to highways and petrol stations and increased risk of childhood leukemia and lymphoma [65]. Raaschou-Nielsen et al. (2018) adjusted for urban development, region, residence type, and other factors, finding a higher risk of AML with benzene exposure [66]. Ghosh et al. (2013) adjusted for maternal age, race/ethnicity, education, SES, and other factors, but they did not clarify if the results changed after these adjustments, acknowledging potential residual confounding [10]. Lee et al. (2022) adjusted for sex, birth year, insurance type, and family income, using smoking rates as a proxy for Environmental Tobacco Smoke (ETS) observing a strong influence of confounders [69]. Kreis et al. (2022) adjusted for socioeconomic position, urbanization, and ionizing radiation, finding minimal impact on associations except for AML [58]. Badaloni et al. (2013) adjusted for parental education level, region, age at diagnosis, and road traffic indicators, finding minimal effect [49]. Brosselin et al. (2009) adjusted for the number of children under 15 years, urbanization, SES, early infections in childhood, smoking, maternal domestic pesticide, and housing type [51]. Symanski et al. (2016) adjusted for the maternal age, maternal race/ethnicity, infant birth weight, and sex, in addition to matching on birth year and month and accounting for census tract effects [61]. In the aforementioned studies, the authors did not explicitly state if the results changed after adjusting for confounders.

##### Broader environmental and demographic factors

Houot et al. (2015) adjusted for age, calendar period, urban unit size, and a deprivation index, finding consistent results across different age groups (≤ 4, 5–9, and 10–14 years) [60]. Malavolti et al. (2023) adjusted for PM_10_, extremely low frequency-magnetic fields (ELF-MF), socio-demographic variables, and land use types, with high residential stability among participants. They did not specify if adjusting for confounders influenced the outcomes [59]. Lavigne et al. (2020) considered maternal age, smoking, infant sex, parity, and SES, and found no overall link between PM_2.5_, NO₂, and childhood cancer. However, they identified a positive association between exposure to ambient ultrafine particles (UFPs) during the first trimester and childhood cancer, including ALL, independent of other pollutants [67]. Heck et al. (2014) adjusted for birth year, maternal race/ethnicity, and other factors, finding benzene exposure may increase leukemia risk [54]. Magnani et al. (2016) adjusted for age, sex, region, parental education and smoking, highlighting the need for leukemia type-specific analyses [52].

In addition, Vinceti et al. (2012) found that adjusting for income increased relative risks (RRs) of childhood leukemia, while adjusting for other pollutants reduced them. PM_10_ was linked to higher RRs in children under 5, but this was lessened when accounting for benzene [48]. Zhong et al. (2023) adjusted for demographic and socioeconomic factors like birth year, sex, maternal race/ethnicity, SES, and other risk factors for childhood ALL. They identified Hispanic ethnicity and higher birthweight as key risk factors and found a borderline association between PM_2.5_ levels and ALL risk among non-Hispanic White children, despite potential residual confounding [56]. Williams et al. (2024) adjusted for birth year, sex, maternal race/ethnicity, and SES, finding a synergistic relationship between green spaces and PM_2.5_ [68]. In those studies authors did not specify if adjusting for confounders influenced the outcomes.

To clarify these findings, we summarized which confounders were considered across the 23 included studies (Table 3). The most common adjusted variables were SES, maternal age, and race/ethnicity, whereas residential mobility, parental smoking and other environmental exposures were less frequently adjusted for. Some studies reported clearly how after adjustment, the effect estimates changed, while others did not.

**Table 3:** Confounders and effect modifiers adjusted for the 23 studies included in the present scoping review

Study	SES/Education	Urbanization	Parental smoking	Maternal age	Race/Ethnicity	Other factors	Reported change after adjustment
Williams et al., 2024	✓	✖	✖	✖	✓	Birth year, sex, NDVI (Green spaces)	Synergistic (PM_2.5_ and NDVI)
Norzaee et al., 2024	✓	✓	✓	✖	✖	Family history of cancer	Positive association
Zhong et al., 2023	✓	✓	✖	✓	✓	Birth weight, delivery mode, pregnancy complications	Borderline effect (PM_2.5_ and non-Hispanic)
Malavolti et al., 2023	✓	✓	✓	✓	✖	ELF-MF, PM_10_, land use	Not specified
Kreis et al., 2022	✓	✓	✖	✓	✖	Ionizing radiation	Minimal change (except AML)
Lee et al., 2022	✓	✓	✓ (ETS proxy)	✖	✖	Insurance type	Strong effect
Mazzei et al., 2022	✓	✓	✖	✖	✖	NO_2_, distance to highway, registry presence	Stronger association
Lavigne et al., 2020	✓	✓	✓	✓	✖	Infant sex, parity	UFP effect remained
Peckham-Gregory et al., 2019	✓	✓	✖	✖	✓	Area-level poverty, birth weight	No effect
Raaschou-Nielsen et al., 2018	✓	✓	✖	✓	✖	Region, residence type, birth order	Positive (AML with benzene)
Seifi et al., 2019	✖	✖	✖	✖	✖	None	No adjustment performed (potential residual confounding)
von Ehrenstein et al., 2016	✓	✓	✖	✓	✓	Birth place, preterm birth	No effect
Magnani et al., 2016	✓	✓	✓	✓	✖	Region	Needed subtype analysis
Symanski et al., 2016	✓	✓	✖	✓	✓	US EPA NATA, modeled data, pollutants	Not specified
Spycher et al., 2015	✓	✓	✖	✖	✖	Ionizing radiation, EMF	No effect
Houot et al., 2015	✓ (Deprivation index)	✓	✖	✖	✖	Age groups, calendar period, road length	Consistent
Heck et al., 2014	✓	✓	✖	✓	✓	-	Positive
Badaloni et al., 2013	✓	✓	✖	✖	✖	Road network, traffic indicators	Minimal change
Ghosh et al., 2013	✓	✖	✖	✓	✓	Parity, insurance type	Not specified
Vinceti et al., 2012	✓ (income)	✓	✖	✖	✖	Pollutant adjustments	Mixed (RR increased with income, decreased with benzene)
Amigou et al., 2011	✓	✓	✓	✖	✖	Housing type	No effect
Brosselin et al., 2009	✓(maternal education)	✓	✓	✖	✖	Housing type and size, and early infections	Not specified
Weng et al., 2009	✓(proxy)	✓	✖	✖	✖	Urbanization index, Year of death	Positive

(✓ = adjusted, ✖ = not adjusted, - = unclear)

## Discussion

This review provides a comprehensive overview of the methodological challenges in studies investigating the association between air pollution exposure and childhood cancer. Our systematic literature search in two different databases, supplemented by manual search in reference lists and in Google Scholar, identified 23 studies that matched the inclusion criteria. Except for four cohort studies, all others used case-control study designs. The majority of these epidemiological studies face recurring methodological challenges that may compromise both internal validity and causal interpretation. Below, we critically map the main challenges and suggest future directions.

An important challenge across studies involves recall and selection bias, particularly in case-control designs. Recall bias appears when parents of affected children report exposures differently from controls, potentially exaggerating observed associations. This bias can be minimized by using objective data sources, such as medical records, registries and biomarkers, in combination with well-structured questionnaires. Prospective study designs can also mitigate recall bias because outcomes are unknown at time of patient enrollment, although these designs are often costly unless based on routinely recorded data [70–72].

Selection bias frequently occurs from hospital-based control participants or incomplete population registries. Future studies should clearly define inclusion and exclusion criteria, apply probability sampling and matching procedures to ensure comparability between cases and controls. Unless these biases are systematically addressed, inconsistencies in reported associations will persist, weakening causal inference and overall conclusions [73].

Moreover, due to the rarity of childhood cancer, studies require very large population-based cohorts or case-control designs to achieve sufficient statistical power. Both strategies, however, can lead to introduce methodological challenges, including incomplete registry information, small sample sizes and non-representative control groups [66]. Such challenges are exacerbated by dependence on parental reporting, residential mobility and limited biomarker data for prenatal and early life exposures [74, 75]. Together, these aforementioned issues complicate exposure assessment and interpretation in studies of childhood cancer.

In addition, exposure measurement remains one of the major methodological challenges in this research field. The absence of standardized and accurate methods for measuring exposure to air pollution in relation to specific health outcomes increases the likelihood of error [76]. Hence, a key recommendation is to standardize exposure assessment methods by integrating multiple approaches (e.g., combining monitoring data with satellite observations) to improve spatial and temporal resolutions. Furthermore, the future use of exposure biomarkers could enhance the environmental estimates and enable more individualized assessment.

The included studies used various exposure assessment methods, ranging from ground-based monitoring to LUR, dispersion models and satellite-based models. Each method has distinct advantages and disadvantages. GIS-based registry studies, which link cancer registry data with LUR models or monitored air pollution concentrations, allow inclusion of large populations and provide cost-effective, spatially-resolved exposure estimates [66]. However, a major limitation of this approach is the lack of information on residential mobility, household exposure or parental behaviours, which can lead to exposure misclassification and limit the ability to adjust for several confounders [74]. These problems become even more complex when exposure data are derived from years not fully overlapping with diagnosis. Consequently, this results in exposure misclassification, particularly in resource-limited settings where updated monitoring data and individual-level information are unavailable, as noted by Seifi et al. 2019 [50].

On the other hand, interview-based studies provide individual-level data on factors such as parental lifestyle, occupational exposures, smoking habits and home environment, contributing to a more comprehensive understanding of potential risk factors [75]. However, such designs are prone to recall bias, are resource intensive, and often suffer from low participation, which limits generalizability. Combining both registry-based and interview-based approaches, as in hybrid designs, could strengthen study methodology. For example, in the ESCALE *“Étude sur les cancers de l’enfant”* (Study on Childhood Cancer), researchers integrated GIS-based registry data with targeted questionnaires, improving validity and reducing limitations inherent in each individual approach [77].

LUR models provide fine spatial resolution (typically 100 m to 1 km) but require extensive GIS or satellite data and strong knowledge of statistical principles. On the other hand, ground-based monitors offer high temporal precision but may not accurately capture individual-level exposure [78]. Proximity-based metrics (e.g., distance to major roads or petrol stations) are simple to implement but provide only crude proxies for exposure and are sensitive to residential mobility, thereby causing non-differential misclassification [51, 79].

Recently, the field has shifted toward hybrid models that combine ground monitoring, dispersion modelling, LUR and satellite data to improve both spatial and temporal resolution and enhance exposure accuracy [80, 81]. Future studies should integrate these advanced models with portable sensors and biomarker validation to achieve individualized exposure estimates [82, 83]. Standardizing exposure assessment by integrating monitoring and satellite data will enhance comparability across studies and reduce measurement error.

Moreover, another key challenge relates to assessing air exposure at various critical periods (e.g., prenatal, early childhood, or cumulative lifetime exposure). Several studies included in our review examined specific exposure windows but were limited by incomplete residential histories, as certain data sources only documented diagnosis date, thereby reducing temporal precision. This issue is particularly important due to the long latency period between exposure and cancer diagnosis.

Future research should focus on integrating residential mobility data, parental occupational histories and high-resolution exposure models to better identify windows of susceptibility. Prospective birth cohorts and linkages with routinely collected administrative data, such as population or cancer registries, provide a valuable approach to improving temporal precision and identifying the most critical exposure periods in a child’s development [84–86].

Reliable and complete outcome data are essential for accurate inference. Most studies obtained cancer data from national or hospital-based registries, although their completeness and quality varied. National cancer registries typically provide high-quality, standardized and population-based data, whereas hospital-based registries or mortality records may lead to underreporting, particularly for deaths occurring outside hospitals that are not systematically registered. Weng et al. (2009), for instance, reported under-registration of childhood leukemia deaths in Taiwan due to missing death certificates [64]. Consequently, this leads to incomplete case registration and complicates the assessment of data quality indicators [87].

In 2023, Lang et al. compared cancer registry data in Rhineland-Palatinate with health insurance data from the Institute for Applied Health Research Berlin (InGef) database. They observed strong agreement across three cancer types (breast, prostate, or lung cancer), including complete information on age at diagnosis, tumor site, radiotherapy and surgical treatment. Although minor differences existed, both data sources were found to complement each other. They suggested that cancer registry data are more useful for studies requiring histopathological information, whereas claims data is preferable for research on comorbidity and hospitalization [88].

Future research should aim to link multiple data sources to increase completeness, validity and depth of information. Likewise, the careful selection of controls from population registries, birth records, or national cohorts enhances representativeness, reduces bias, improves comparability, minimizes confounding variables, and strengthens statistical power. In summary, the availability of high-quality cancer registry data is essential in the planning for cancer control. Otherwise, these data risk being underutilized or misleading.

Most studies adjusted for socioeconomic status (SES), residence type and urbanization level, finding minimal or no impact on effect estimates, suggesting that these factors may not strongly influence childhood cancer risk. However, residual confounding remains a concern. For instance, Mazzei et al. (2022) found stronger associations after adjustment, highlighting the complexity of these associations [63]. The lack of clarity in reporting post-adjustment results, as seen in Ghosh et al. (2013) and Malavolti et al. (2023), underscores the need for more transparent reporting practices [10, 59]. However, Seifi et al. (2019) relied solely on unadjusted odds ratios, potentially overestimating the observed association between PM_10_ and childhood cancer [50].

Furthermore, only a few studies in our review clearly examined effect modification. For example, Spycher et al. (2015) and Kreis et al. (2022) adjusted for background ionising radiation to address potential confounding, rather than to test for interaction effects [57, 58]. Similarly, Spix et al. (2017) conducted an ecological study in Germany and reported inconsistent associations between background gamma radiation and childhood cancer [89]. Although, background radiation has been proposed as a potential interacting exposure- because Ionizing radiation is a well-established carcinogen, including gamma radiation, is well-established human carcinogen according to IARC and American Cancer Society- it could theoretically enhance susceptibility to other environmental agents-no study in this review formally evaluated it as an effect modifier [90, 91]. Likewise, no study assessed interaction with radon, revealing a notable research gap in research on combined environmental exposures. Other plausible effect modifiers, such as sex or age at exposure, were not the primary focus of this review, which aimed to map broader methodological challenges across the field rather than to comprehensively evaluate every potential modifier. Future studies should not only address confounding but also adopt modern statistical approaches, such as multi-exposure modelling, Bayesian hierarchical models or causal inference frameworks that can evaluate interactions among multiple pollutants. Such approaches would better reflect the multifactorial etiology of childhood cancer and enhance causal interpretation.

Across the 23 included studies, results were mixed. Many reported positive associations between air pollutant exposure (e.g., traffic-related air pollution, benzene, PM_2.5_) and childhood cancers [10, 48, 51–61, 64–69]. In contrast, others found no or weak associations [10, 49, 62, 63]. These inconsistencies are likely due to methodological challenges discussed above, particularly those related to exposure assessment, study design and data quality, rather than the absence of true associations. Standardized research methods and large, multi-country studies are needed to enable international data pooling, which is particularly important for studying rare outcomes like childhood cancer.

Despite methodological challenges, most studies showed a consistent positive association between air pollution exposure and childhood cancer, especially for childhood leukaemia and traffic-related benzene exposure, across various study designs and populations. This consistency unlikely due to chance alone, supporting the hypothesis that air pollution may be a potential risk factor for childhood cancers. Oppositely, associations were less consistent for other pollutants and non-leukaemia cancers, possibly due to methodological differences, incomplete detection of critical exposure windows, residual confounding and limited statistical power for rare outcomes. Moreover, the biological mechanism linking benzene exposure to leukaemia is stronger than that for solid tumours, which may partly explain these differences [92].

### Quality and strength of the evidence

Despite the absence of a formal risk-of-bias assessment, consistent patterns were observed across the included studies and can be highlighted. In general, registry- and record-based studies were less prone to recall bias and demonstrated greater internal validity than interview-based designs. However, discrepancies in exposure assessment methods (e.g., proximity models, LUR, dispersion models and satellite data) limited the comparability of finding across studies. Evidence for benzene and traffic-related exposures was most consistent in relation to ALL, while evidence for PM_2.5_ and NO_2_, as well as for brain tumors and other childhood cancers, remains inconclusive. In summary, the overall strength of evidence can be considered moderate for ALL and traffic-related pollutants, but weaker for other cancer types and pollutants, emphasizing the need for standardized and high-quality studies.

Understanding the methodological and environmental challenges involved in studying this association is of paramount importance, as it may assist future researchers design more robust studies that proactively account for potential epidemiological challenges and are better equipped to overcome them.

### Strengths

To our knowledge, this is the first scoping review to summarise the possible methodological challenges in epidemiological studies investigating the association between air pollution exposure and childhood cancer. Given that childhood cancer and air pollution are major global health challenges contributing to increased mortality and morbidity, several studies have addressed these topics individually but not collectively, as in our review.

An important strength of scoping reviews is that they provide a rigorous and transparent framework for mapping existing areas of research within a relatively short timeframe, compared to comprehensive systematic reviews. In our analysis, we were able to identify gaps in the evidence base and summarize key research findings. By presenting results in an accessible and condensed format, this review supports policymakers and future researchers in effectively utilizing the findings to guide policy development, research priorities and public health interventions [93].

### Limitations

Although this paper aimed to summarize a broad range of published literature on the association between air pollution exposure and childhood cancer, we did not assess the methodological quality of the included studies. Our conclusions are based on the reported content of the included studies rather than their intrinsic quality.

Additionally, the accuracy of our review is constrained by its dependence on information reported in published papers, which may lack sufficient details on confounder adjustment or bias management. Furthermore, our search was limited to two major databases (PubMed and Web of science) and Google Scholar, and did not include Scopus or Embase. As a result, we may have missed some relevant studies indexed exclusively in these databases or in the grey literature. To mitigate this limitation, we additionally screened the reference lists of all included articles to identify any studies that were not captured through our database search. A further limitation is that we defined childhood based on the UNCRC cutoff of 0–18 years, and therefore exclude studies that did not report estimates separately for this age range. In some countries, childhood cancer is defined up to 19 years of age, and relevant studies such as these by Hvidtfeldt et al. (2020) and Erdmann et al. (2022) were excluded for these reasons [34–39]. This difference in age definition may have resulted in the exclusion of some eligible populations and limited the international representation in our review.

## Conclusion

Although several epidemiological studies have investigated the association between air pollution exposure and childhood cancer risk, numerous methodological challenges persist, limiting causal inference. Further studies should prioritize improvements in exposure assessment methods by combining standardized and integrated methods, such as linking satellite data with personal monitoring devices and biomarkers. Registry linkage and the use of insurance data resources can further enhance outcome verification. Given the rarity of childhood cancers, designing large, multicenter international pooled studies, particularly in high-exposure settings and low- and middle-income countries is crucial. Furthermore, studies must focus on relevant exposure time windows, especially during prenatal and early-life periods. Finally, the application of advanced statistical methods (e.g., Bayesian hierarchical models and causal inference frameworks) is needed to more effectively address multiple pollutants and confounders, thus improving analytical robustness.

In summary, enhancing the methodological quality of research in this field will provide stronger evidence to guide policymakers and public health interventions aimed at protecting children from the adverse health effects of air pollution.

## Data Availability

All data analysed in this scoping review are based on previously published studies, which are cited and referenced in the manuscript. No new datasets were generated.

## Appendix

### Search prompts for literature databases

A) MEDLINE via PubMed.

**Table:**

#1	(((((((((“Air Pollutants“[Mesh]) OR “Air Pollution“[Mesh]) OR “Environmental Exposure“[Mesh]) OR “Particulate Matter“[Mesh]) OR “Vehicle Emissions“[Mesh]) OR “Traffic-Related Pollution“[Mesh]) OR “Nitrogen Oxides“[Mesh]) OR “Ozone“[Mesh]) OR “Sulfur Dioxide“[Mesh]) OR “Fossil Fuels“[Mesh]
#2	Environment*[Title/Abstract] AND (expos*[Title/Abstract] OR toxic*[Title/Abstract] OR contaminat*[Title/Abstract])
#3	particulate*[Title/Abstract] AND (matter[Title/Abstract] OR air[Title/Abstract])
#4	(smog[Title/Abstract] OR fume*[Title/Abstract] OR exhaust*[Title/Abstract] OR diesel[Title/Abstract]) AND air[Title/Abstract]
#5	(vehicle[Title/Abstract] OR traffic[Title/Abstract]) AND (emission*[Title/Abstract] OR pollut*[Title/Abstract])
#6	(air[Title/Abstract] OR ambient[Title/Abstract]) AND (pollut*[Title/Abstract] OR quality[Title/Abstract])
#7	fossil fuel*[Title/Abstract]
#8	“PM10“[Title/Abstract] OR “PM2.5“[Title/Abstract]
#9	SO2[Title/Abstract] OR NO2[Title/Abstract] OR O3[Title/Abstract] OR CO[Title/Abstract]
#10	#1 OR #2 OR #3 OR #4 OR #5 OR #6 OR #7 OR #8 OR #9
#11	“Leukemia“[MeSH Terms] OR “Lymphoma“[MeSH Terms] OR “Nervous System Neoplasms“[MeSH Terms]
#12	Leukemia*[Title/Abstract]
#13	Leucocythemia*[Title/Abstract]
#14	Leukemia*[Title/Abstract]
#15	Lymphoma*[Title/Abstract]
#16	(“central nervous system“[Title/Abstract] OR cns[Title/Abstract]) AND (neoplasm*[Title/Abstract] OR lymphoma*[Title/Abstract] OR tumor*[Title/Abstract] OR tumour*[Title/Abstract])
#17	#11 or #12 or #13 or #14 or #15 or #16
#18	#10 and #17
#19	Infan* OR newborn* OR new-born* OR perinat* OR neonat* OR baby OR baby* OR babies OR toddler* OR minors OR minors* OR boy OR boys OR boyfriend OR boyhood OR girl* OR kid OR kids OR child OR child* OR children* OR schoolchild* OR schoolchild OR school child[tiab] OR school child*[tiab] OR adolescen* OR juvenil* OR youth* OR teen* OR under*age* OR pubescen* OR pediatrics[mh] OR pediatric* OR paediatric* OR peadiatric* OR school[tiab] OR school*[tiab] OR prematur* OR preterm*
#20	#18 and #19
#21	(Case-Control Studies[MESH: NOEXP] OR Matched-Pair Analysis[MESH: NOEXP] OR “case control“[TIAB])
#22	cohort studies[MESH: NOEXP] OR longitudinal studies[MESH: NOEXP] OR follow-up studies[MESH: NOEXP] OR prospective studies[MESH: NOEXP] OR retrospective studies[MESH: NOEXP] OR cohort[TIAB] OR longitudinal[TIAB] OR prospective[TIAB]
#23	#21 or #22
#24	#20 AND #23
#25	(“2009“[Date - Publication] : “3000“[Date - Publication])
#26	#24 and #25

B) Web of Science.

**Table:**

#1	TS=(“environment*” NEAR/6 (“expos*” OR “toxic*” OR “contaminat*”))
#2	TS=(“particulate*” NEAR/6 (“matter” OR “air”))
#3	TS=((“smog” OR “fume*” OR “exhaust*” OR “diesel”) NEAR/6 “air”)
#4	TS=((“vehicle” OR “traffic”) NEAR/6 (“emission*” OR “pollut*”))
#5	TS=((“air” OR “ambient”) NEAR/6 (“pollut*” OR “quality”))
#6	TS=(“fossil fuel*”)
#7	TS=((“PM10” OR “PM2.5”))
#8	TS=((“SO2” OR “NO2” OR “O3” OR “CO”))
#9	#8 OR #7 OR #6 OR #5 OR #4 OR #3 OR #2 OR #1
#10	TS=((“central nervous system” OR cns) NEAR/6 (neoplasm* OR lymphoma* OR tumor* OR tumour*))
#11	TS=(Lymphoma* OR Leukemia* Leucocythemia* Leukemia*)
#12	#11 OR #10
#13	TS=(Infan* OR newborn* OR new-born* OR perinat* OR neonat* OR baby OR baby* OR babies OR toddler* OR minors OR minors* OR boy OR boys OR boyfriend OR boyhood OR girl* OR kid OR kids OR child OR child* OR children* OR schoolchild* OR schoolchild OR school child OR school child* OR adolescen* OR juvenil* OR youth* OR teen* OR under-age* OR pubescen* OR pediatrics OR pediatric* OR paediatric* OR peadiatric* OR school OR school* OR prematur* OR preterm*)
#14	#13 AND #12 AND #9
#15	#13 AND #12 AND #9 Timespan: 2009-01-01 to 2024-06-05

## Abbreviations

IARC: International Agency for Research on Cancer
PM: Particulate Matter
ALL: Acute Lymphoblastic Leukemia
AML: Acute Myeloid Leukemia
WHO: World Health Organization
SES: Socioeconomic Status
PRISMA-ScR: Preferred Reporting Items for Systematic Reviews and Meta-Analyses—Extension for Scoping Reviews
MeSH: Medical Subject Heading
CNS: Central Nervous System
NO_2_: Nitric Dioxide
POM: Polycyclic Organic Matter
PAHs: Polycyclic Aromatic Hydrocarbons
UFPs: Ultrafine particles
MODIS: Moderate Resolution Imaging Spectroradiometer
MISR: Multi-angle Image SpectroRadiometer
SeaWiFS: Sea-viewing Wide Field-of-view Sensor
NDVI: Normalized Difference Vegetation Index
GIS: Geographic Information System
ALAN: Artificial Light At Night
ELF-MF: Extremely Low Frequency -Magnetic Fields
SNC: Swiss National Cohort
LUR: Land Use Regression
EPA: Environmental Protection Agency
NATA: National-Scale Air Toxics Assessment
SCCR: Swiss Childhood Cancer Registry
COPERT IV: COmputer Programme to calculate Emissions from Road Transport
PSD: Petrol station density
CPC: Chinese Petroleum Corporation
FPCC: Formosa Petrochemical Corporation
NATA: National-Scale Air Toxics Assessment
CARB: The California Air Resources Board
POGONIS: Paediatric Oncology Group of Ontario Networked Information System
NHIS: National Health Insurance Service
ETS: Environmental Tobacco Smoke
